# Right vertical infra-axillary thoracotomy for surgical repair of paediatric ventricular septal defect: a propensity score matched cohort study

**DOI:** 10.1093/icvts/ivaf153

**Published:** 2025-06-27

**Authors:** Hongbo Luo, Qin Zhou, Honggen Wu, Jingjing Zhu, Jin Shentu, Guocheng Shi, Wenxuan Dai, Huiwen Chen

**Affiliations:** Department of Cardiovascular Surgery, The Affiliated Hospital of Guizhou Medical University, Guiyang, Guizhou Province, China; Guizhou Branch of Shanghai Children’s Medical Center, Guizhou Province, China; Department of Anesthesiology, Shanghai Children’s Medical Center, Shanghai Jiao Tong University School of Medicine, Shanghai, China; Department of Cardio-Thoracic Surgery, Congenital Heart Center, Shanghai Children’s Medical Center, Shanghai Jiao Tong University School of Medicine, Shanghai, China; Department of Cardio-Thoracic Surgery, Congenital Heart Center, Shanghai Children’s Medical Center, Shanghai Jiao Tong University School of Medicine, Shanghai, China; Department of Cardio-Thoracic Surgery, Congenital Heart Center, Shanghai Children’s Medical Center, Shanghai Jiao Tong University School of Medicine, Shanghai, China; Department of Cardio-Thoracic Surgery, Congenital Heart Center, Shanghai Children’s Medical Center, Shanghai Jiao Tong University School of Medicine, Shanghai, China; Department of Cardio-Thoracic Surgery, Congenital Heart Center, Shanghai Children’s Medical Center, Shanghai Jiao Tong University School of Medicine, Shanghai, China; Department of Cardiovascular Surgery, The Affiliated Hospital of Guizhou Medical University, Guiyang, Guizhou Province, China; Guizhou Branch of Shanghai Children’s Medical Center, Guizhou Province, China; Department of Cardio-Thoracic Surgery, Congenital Heart Center, Shanghai Children’s Medical Center, Shanghai Jiao Tong University School of Medicine, Shanghai, China

**Keywords:** minimal invasive surgery, cardiothoracic surgery, ventricular septal defect, congenital heart disease, pediatrics

## Abstract

**OBJECTIVES:**

The goal of this study was to evaluate the feasibility and learning curve of a right vertical infra-axillary thoracotomy (RVIAT) in the surgical closure of a ventricular septal defect (VSD).

**METHODS:**

Clinical outcomes in paediatric patients (<18 years) undergoing VSD operations between 2018 and 2021 in 2 tertiary hospitals were reviewed retrospectively. After 1:1 propensity score matching, patients undergoing an RVIAT were compared with those undergoing a median sternotomy (MS). The learning curve that reflected the number of cases needed to achieve technical proficiency was measured using total operating time as a metric and was evaluated using a risk-adjusted cumulative sum analysis.

**RESULTS:**

Of the 3515 eligible patients, 2183 (62%) underwent an MS and 1332 (38%) underwent an RVIAT. After matching, 797 cases in the RVIAT and MS groups were recorded, respectively. Propensity weighting produced an excellent balance in patient baseline characteristics including age, weight, and VSD subtypes. There was no between-group difference in postoperative rhythm disturbances (0.6% vs 1.1%; *P *= 0.83), significant residual VSD (0.1% vs 0.4%, *P *= 0.62), and reoperation within 60 days postoperatively (0.1% vs 0.9%, *P *= 0.07). The RVIAT provided better cosmesis (satisfactory score: 9.21 ± 0.06 points vs 6.98 ± 1.17 points; *P *< 0.001), shorter median length of hospital stay (5.5 days vs 8.0 days, *P *< 0.001), and lower cost (8513.3 ± 3193.2 USD vs 9222.3 ± 2504.9 USD; *P *< 0.001). The surgeons could conquer the early learning phase of the RVIAT after performing a mean of 41 operations.

**CONCLUSIONS:**

A RVIAT can combine good outcomes with favourable cosmesis in VSD repair, and sufficient exposure to RVIAT procedures is crucial for proficiency.

## Graphical abstract

**Figure:**
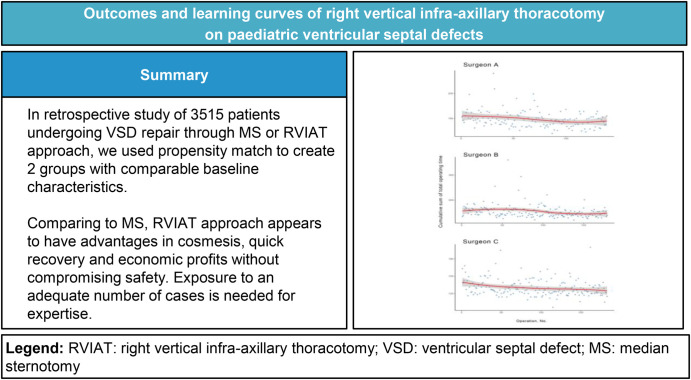

## INTRODUCTION

A median sternotomy (MS) remains the gold standard procedure for congenital heart surgery owing to the ease of cannulation and the excellent exposure^1^. However, visible scar and thoracic deformities stigmatize and trigger psychological distress in patients and their families^2^^,^^3^. The American Heart Association has included psychological health within the Life’s Essential approach for delivering optimal cardiovascular care^4^. Accordingly, the surgical community should be advocated to pursue minimally invasive approaches without compromising safety to provide psychological support to this patient population.

Currently, a right vertical infra-axillary thoracotomy (RVIAT) has gained popularity because it can offer a cosmetically more appealing incision, and good results have been reported in a diverse set of congenital heart disease patients^5–10^. Nevertheless, a consensus that an RVIAT is a reasonable and safe alternative to MS to correct a ventricular septal defect (VSD) has not been achieved; therefore, discretionary triage to the RVIAT approach occurs among centres. This observation can be partially reflected in the fact that minimally invasive approaches for VSD closure compromise only about one-fifth of all reported procedures^11^. A main reason may be that many centres are faced with the dilemma of how to develop the RVIAT, particularly in cases of unfavourable patient size or VSD subtypes, and how to have surgeons overcome the learning curve for this technique.

Robust data comparing the RVIAT and the MS for VSD repair are scarce. In addition, the learning curve effect of the RVIAT has not been adequately studied. More evidence of feasibility, safety, and especially the learning curve is needed before further generalization of the routine use of RVIAT for VSD repair can be promoted. This multicentre matched control study was designed to address these knowledge gaps by analysing outcomes of RVIAT for VSD closure in comparison to repair through an MS, and to assess specifically the learning curve for the RVIAT.

## PATIENTS AND METHODS

### Participants

**Figure 1.**
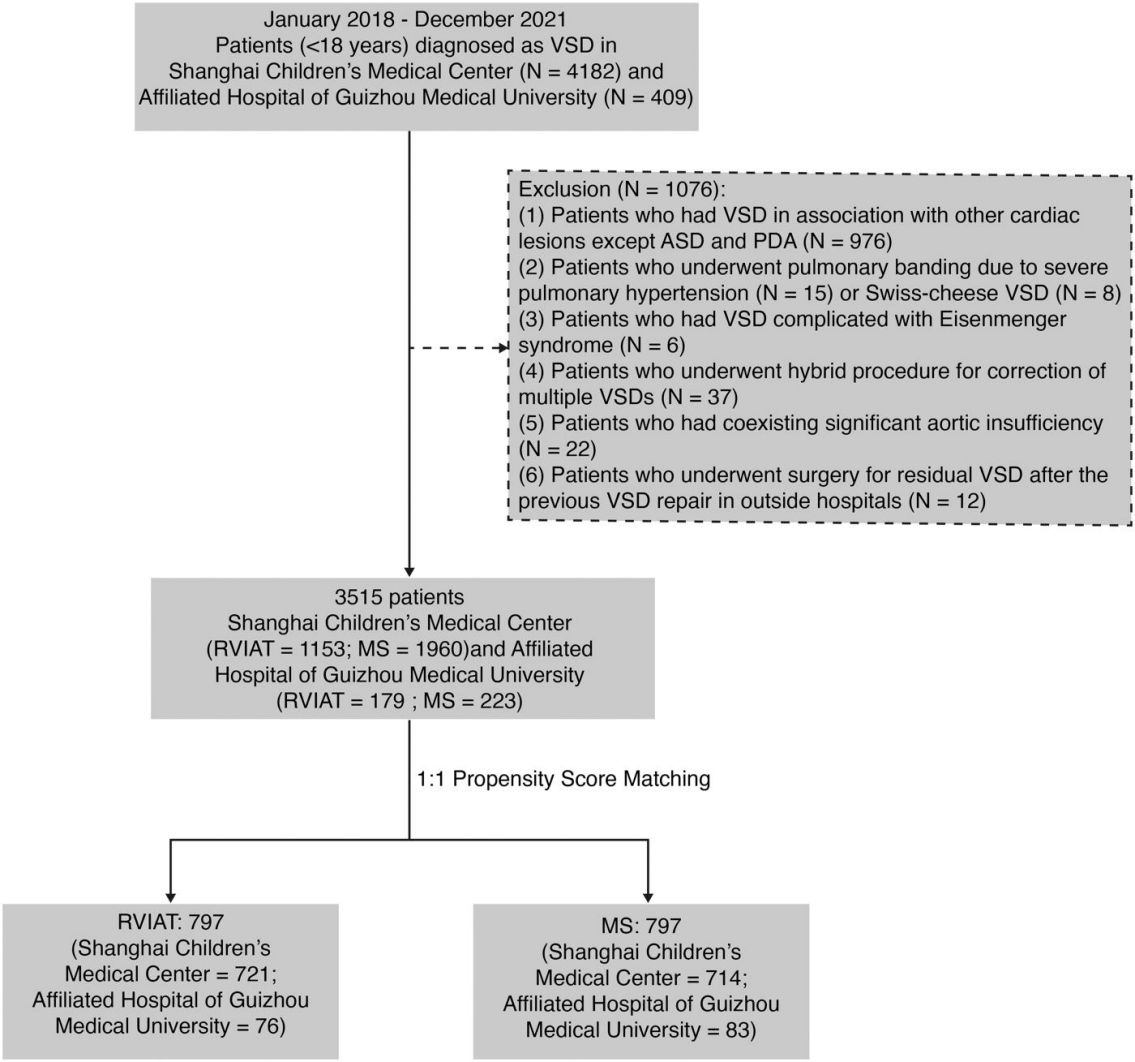
Study flow chart. ASD: atrial septal defect; MS: median sternotomy; PDA: patent ductus arteriosus; RVIAT: right vertical infra-axillary thoracotomy; VSD: ventricular septal defect.

Patients (<18 years of age) undergoing surgical repair of a VSD in Shanghai Children’s Medical Center and the Affiliated Hospital of Guizhou Medical University in 2018 to 2021 were included consecutively, and the data were reviewed retrospectively. Exclusion criteria were patients (i) who had VSD in association with other cardiac lesions except an atrial septal defect and patent ductus arteriosus; (ii) who underwent pulmonary banding due to severe pulmonary hypertension or Swiss-cheese VSD; (iii) who had VSD with Eisenmenger syndrome and were not qualified for VSD closure; (iv) who underwent a hybrid procedure for correction of multiple VSDs; (v) who had coexisting significant aortic insufficiency; and (vi) who underwent surgery for residual VSD after a previous VSD repair in an outside hospital (**Figure 1**).

### Ethical statement

This study was approved by the Shanghai Children’s Medical Center institutional review board (SCMCIRB-K2023153-1). Written informed consent was provided by all participants or their parents. The collection and storage of data were conducted in accordance with the WMA Declaration of Taipei. The establishment and ongoing use of databases were approved and monitored by a research ethics committee.

### Surgery

**Figure 2.**
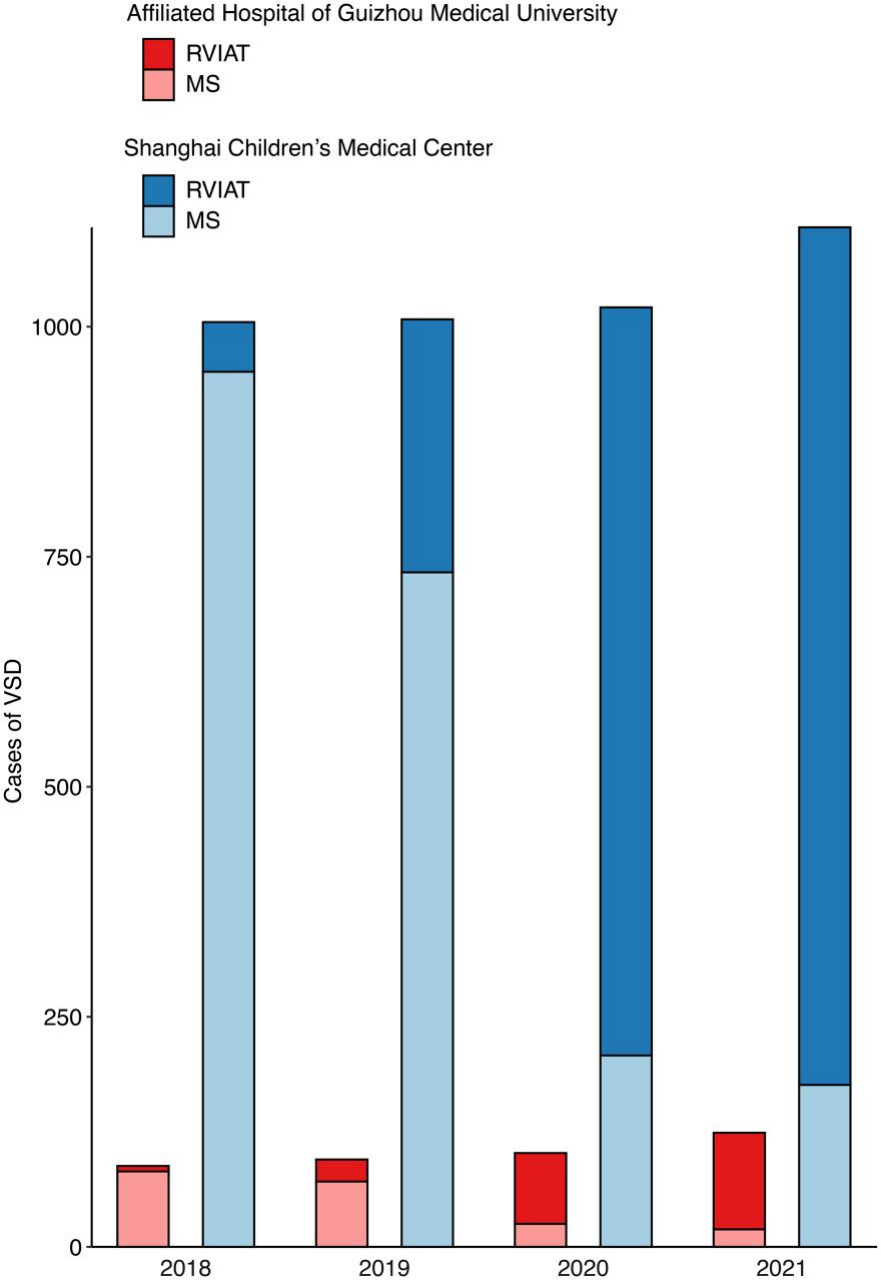
Changes in number of VSD procedures performed using RVIAT versus MS from 2018 to 2021 in two centres. MS: median sternotomy; RVIAT: right vertical infra-axillary thoracotomy; VSD: ventricular septal defect.

An RVIAT for VSD closure was first introduced in January 2018 in the 2 centres (**Figure 2**). Three attending surgeons (surgeon A and C were from Shanghai Children’s Medical Center; surgeon B was from the Affiliated Hospital of Guizhou Medical University) were early adopters of this procedure. During the later study period, either of the 3 surgeons would be an assistant and teach the resident surgeons. Thus, the resident surgeons could benefit from the mentorship of these 3 surgeons. A detailed institutional standard of procedure for this approach is provided in Supplementary Table S1. Specifically, it should be clarified that each of the 3 aforementioned attending surgeons is experienced and had performed >500 VSD operations through the MS approach before January 2018.

A film of the RVIAT procedure is shown in **Video 1**. Briefly, patients received endotracheal intubation and were positioned in a 45° left lateral position. All patients underwent central cannulation, and one-lung ventilation was used in all of the patients of >6 months of age to provide satisfactory exposure. A midaxillary skin incision of 3–5 cm was made overlying the fourth intercostal space. The thymus was resected, and the pericardium was opened 1.5–2 cm anterior to the phrenic nerve. Five stay sutures were placed along the margins of the pericardium and fixed to the surrounding drapes for better exposure. A second midaxillary skin incision of 0.5 cm was made in the seventh intercostal space. This site was where the intravenous cannula would be tunnelled through the skin. Extracorporeal circulation was established following direct central arterial and bicaval cannulation. After a right atriotomy, 3 traction sutures were placed for better exposure of the VSD, including 2 placed on the tricuspid valve annulus and 1 placed 2 cm away from the defective rim on the right side of the ventricular septum. VSD closure was performed using a running suture. As for doubly committed subarterial VSD, we longitudinally opened the pulmonary trunk and closed the defect with a patch. An inlet muscular VSD (<3 mm) was closed directly using interrupted pledget stitch.

### Data collection and outcomes

Review of the medical records, including diagnostic reports (e.g., echocardiogram, cardiac tomography angiography), operative notes, and outpatient records, was performed manually by a 3-year fellow surgeon who was blinded to the data analyses to obtain demographic information [age, sex, weight, height, operation era, preoperative N-terminal pro-B-type natriuretic peptide (NT-proBNP), history of recurrent pneumonia, anatomical characteristic of VSD, coexisting lesions, surgical details [cannulation type (central vs peripheral), total operating time, cardiopulmonary bypass, and aortic cross-clamping (AXC) time, ventilatory strategy (one- vs two-lung ventilation)], and follow-up information. VSD was classified into perimembranous, doubly committed subarterial, and muscular defect^12^. Perimembranous VSD was further subdivided into centrally located (defect opens centrally in ventricular base), inlet (defect extends to inlet behind septal leaflet of the tricuspid valve), and outlet extension (defect extends to ventriculo-infundibular fold)^13^. Muscular VSD was subdivided into inlet, midseptal, and apical defects. Multiple VSD was defined as the presence of both perimembranous and muscular defects. Residual significant VSD was defined as a defect of >2 mm noted on the echocardiogram during the operation or at the follow-up.

The primary composite end points included postoperative rhythm disturbances requiring temporary cardiac pacing, residual significant VSD, or reoperation due to pericardial effusion or bleeding within 60 postoperative  days. The key secondary end points included length of hospital stay, hospitalization cost, and cosmetic satisfaction at 1 year postoperatively (measured by the participants or parents using a 10-point scale with higher scores indicating greater satisfaction) (Supplementary Table S2). Other secondary end points included cardiopulmonary bypass and AXC times, duration of ventilation, duration of chest tube drainage, cardiac intensive care unit stay, and readmission due to pulmonary complications within 6 months postoperatively.

### Follow-up

Patients were required to attend a structured examination at 1, 6, and 12 months postoperatively and then annually. Additionally, patients had access to timely online consultations via the haodf network (https://www.haodf.com), which is the biggest online health consultant service platform in China. Echocardiograms, electrocardiograms, and chest rays were routinely performed at each follow-up visit. The final date of follow-up for data collection was 31 January 2023.

### Statistics

Parametric distribution of the data was tested using the Shapiro–Wilk test, and continuous data were summarized using the mean ± standard deviation (SD) or median [interquartile range (IQR)] if skewed. Categorical variables were reported as counts and percentages. Comparisons between the RVIAT and MS groups were performed using the pairwise *t* test or the Wilcoxon test for continuous variables or the McNemar test for categorical variables as appropriate.

To adjust for the non-random assignment of the procedure (RVIAT vs MS), a propensity score matching analysis was performed using a greedy algorithm and nearest-neighbour approach with a caliper width of 0.1 of the SD of the logit of the propensity score. The propensity score was estimated using a multivariable logistic regression, with the type of operation as the dependent variable and age, weight, height, sex, VSD subtype, NT-proBNP levels, coexisting cardiac lesions, preoperative recurrent pneumonia, and hospitals as covariates. Standardized mean differences for all covariates were calculated before and after matching, with 10% or more considered indicative of imbalance.

To investigate the learning curve effect of the total operating time, surgeons who had performed >100 consecutive RVIAT procedures since their first case were included. Overall, 501 of 1332 cases in the RVIAT group performed by 3 surgeons were identified (surgeons A, B, C: 139, 179, 183 cases, respectively). Learning curve effects were analysed using the risk-adjusted cumulative sum (RA-CUSUM) method^14^ and were constructed following 2 steps: (i) an estimated total operating time was generated using a multivariable linear regression model, with the dependent variables (age, weight, height, sex, VSD subtype, coexisting lesions, preoperative NT-proBNP, and history of recurrent pneumonia preoperatively) included in the model; (ii) the deviations between the actual total operating time and the estimated total operating time were accumulated. The CUSUM curve reflected the deviation between the actual and the expected total operating times, wherein the ascending curve indicated a longer actual total operating time than the expected total operating time, and the descending curve indicated a shorter actual total operating time than the expected total operating time. The inflexion point when the RA-CUSUM score began to stabilize or decrease was estimated using the broken-line model^15^.

All tests were two-sided, and a *P*-value <0.05 was considered statistically significant. Statistical analyses were completed in R (version 4.1.3) (R Foundation for Statistical Computing, Institute for Statistics and Mathematics, Vienna, Austria).

## RESULTS

### Demographics

A total of 3515 patients were included, with 1332 in the RVIAT group [mean (SD) age: 30.9 (21.7) months] and 2183 in the MS group [mean (SD) age: 17.0 (23.4) months]. After matching, 797 patients from the RVIAT group were compared with 797 control patients in the MS group. Between-group comparisons demonstrated no difference in age (RVIAT: 30.7 ± 21.4 months vs MS: 31.9 ± 27.5 months; *P *= 0.15, SMD= -0.05), weight (13.0 ± 4.8 kg vs 13.1 ± 6.8 kg; *P *= 0.72, SMD = −0.014), height (71.5 ± 14.2 cm vs 74.0 ± 19.2; *P *= 0.68, SMD = 0.02), VSD subtype (*P*= 0.71, SMD = −0.15), and preoperative NT-proBNP [RVIAT: 91 (IQR, 37.0–177.0) pg/ml vs MS: 86.0 [IQR, 35.0–287.0] pg/ml; *P *= 0.34, SMD = 0.12). **Table 1** shows the detailed demographic and clinical characteristics of the pre- and post-matched cohort at baseline.

**Table 1.** Baseline characteristics before and after propensity score matching

	Before matching			After matching				
	RVIAT (*n* = 1332)	MS (*n* = 2183)	*P*-value	SMD	RVIAT (*n* = 797)	MS (*n* = 797)	*P* value	SMD
Age, mean ± SD, months	30.90 ± 21.70	17.02 ± 23.41	<0.001^a^	0.62	30.7 ± 21.4	31.9 ± 27.5	0.15^a^	−0.05
Male (%)	839 (63.0)	1303 (59.7)	0.03^b^	0.07	448 (56.2)	450 (56.5)	0.72^b^	−0.01
Weight, mean ± SD, kg	13.02 ± 4.84	9.30 ± 6.25	<0.001^a^	0.66	12.97 ± 4.75	13.07 ± 6.76	0.68^a^	0.02
Height, mean ± SD, cm	89.62 ± 16.58	74.43 ± 19.06	<0.001^a^	0.85	71.49 ± 14.24	73.98 ± 19.21	0.61^a^	−0.15
Preoperative NT-proBNP, median [IQR], pg/ml	89 [36, 133]	105 [43, 313]	<0.001^c^	−1.20	91 [37, 177]	86 [35, 287]	0.34^c^	0.12
Recurrent pneumonia preoperatively (%)	97 (7.3)	233 (10.7)	<0.001^b^	−0.12	60 (7.5)	64 (8.0)	0.77^b^	−0.02
VSD subtype			<0.001^b^				0.72^b^	
Perimembranous VSD^d^ (%)	987 (74.1)	1390 (63.7)		0.23	552 (69.2)	539 (67.6)		0.04
Doubly-committed VSD (%)	312 (23.4)	714 (32.7)		−0.21	223 (28.0)	232 (29.1)		−0.03
Multiple VSD (%)	33 (2.8)	79 (3.6)		−0.07	22 (2.8)	26 (3.3)		−0.03
Association of other cardiac lesions								
ASD (%)	199 (14.9)	371 (17.0)	0.01^b^	−0.06	168 (21.1)	169 (21.2)	0.13^b^	0.00
PDA (%)	76 (5.7)	223 (10.2)	<0.001^b^	−0.17	41(5.1)	47 (5.9)	0.18^b^	−0.03
Centre			0.03^b^				0.23^b^	
Affiliated Hospital of Guizhou Medical University	179 (13.4)	223 (10.2)		−0.04	76 (9.5)	83 (10.4)		−0.03
Shanghai Children’s Medical Center	1153 (86.6)	1960 (89.8)		0.04	721 (90.5)	714 (89.6)		0.03

a *P* value was generated by pairwise *t*-test.

b *P* value was generated by McNemar test.

c *P* value was generated by pairwise Wilcoxon test.

d Perimembranous ventricular septal defect was further subcategorized into centrally located (before matching: *n* = 1105; after matching: *n* = 533), inlet extension (before matching: *n* = 934; after matching: *n* = 395), outlet extension (before matching: *n* = 322; after matching: *n* = 154) and misaligned (before matching: *n* = 16; after matching: *n* = 9).

ASD: atrial septal defect; IQR: interquartile range; MS: median sternotomy; PDA: patent ductus arteriosus; RVIAT: right vertical infra-axillary thoracotomy; SD: standard deviation; SMD: standardized mean difference; VSD: ventricular septal defect.

### Outcomes

No deaths occurred in the entire cohort (*N* = 3515). In the matched cohort, follow-up was completed in 784 (98.6%) patients with a median of 32.8 (IQR, 24.6–43.0) months in the RVIAT group and 779 patients in MS group (97.7%) with a median of 40.9 (IQR, 27.9–49.1) months. Eleven patients in the RVIAT group came from other countries or regions (North America: 5, Middle east: 3, Japan: 2, India: 1) and were followed in their local hospitals.

After matching, there were no statistical differences in the primary outcomes between the RVIAT and MS groups despite an overall longer AXC time observed in the RVIAT group (**Table 2**). Temporary epicardial pacing was required in 5 patients in the RVIAT and 9 in the MS group due to II° atrioventricular block, and the rhythm returned to normal within a median of 2 (IQR, 1–4) days postoperatively. None in the 2 groups developed III° atrioventricular block and required permanent cardiac pacing. A total of 6 patients (all in the MS group) were readmitted to the hospital for reoperations due to pericardial effusion within postoperative 60 days. Patients in the RVIAT group had shorter length of postoperative stays and shorter durations of ventilation as well as chest drainage (**Table 2**). The total health-care costs in the RVIAT group were also lower than those in the MS group (8513.3 ± 3193.2 USD vs 9222.3 ± 2504.9 USD; *P *< 0.001). Parent- or patient-reported cosmetic satisfaction was significantly greater in the RIVAT group than in the MS group (9.21 ± 0.06 vs 6.98 ± 1.17 points, *P *< 0.001; Supplemental Table S3).

**Table 2.** Between-group comparisons in outcomes

Primary composite end points	RVIAT (*n* = 797)	MS (*n* = 797)	*P* value
Death	0 (0.0)	0 (0.0)	NA
Rhythm disturbances requiring temporary cardiac pacing (%)	5 (0.6)	9 (1.1)	0.10^a^
Residual significant VSD (%)	1 (0.1)	3 (0.4)	0.44^a^
Reoperation due to pericardial effusion or bleeding (%)	1(0.1)^b^	7 (0.9)^c^	0.06^a^

**Perioperative in-hospital data**	**RVIAT (*n* = 797)**	**MS (*n* = 797)**	***P* value**

Total operating time, min	143.83 ± 23.10	127.45 ± 19.53	<0.001^d^
CPB time, mean ± SD, min	61.66 ± 31.47	53.85 ± 21.11	<0.001^d^
AXC time, mean ± SD, min	35.15 ± 16.68	29.26 ± 12.02	<0.001^d^
Duration of ventilation, median [IQR], h	5.52 [4.37, 7.47]	5.72 [4.48, 8.47]	0.002^e^
Duration of thoracic drainage, median [IQR], days	3.00 [2.50, 4.00]	3.50 [3.00, 4.00]	<0.001^e^
CICU stay, median [IQR], h	19.00 [15.74, 21.65]	19.48 [15.91, 33.25]	<0.001^e^
Length of hospital stay, median [IQR], days	5.50 [5.50, 7.50]	8.00 [7.00, 10.00]	<0.001^e^
Sternal non-union (%)	0 (0)	5 (0.6)	0.03^a^
Wound infection (%)	4 (0.5)	11 (1.3)	0.02^a^
Operation cost, mean ± SD, US dollars	2315.8 ± 512.4	2355.3 ± 527.8	0.13^d^
Postoperative cost, mean ± SD, US dollars	5183.2 ± 1074.6	5975.0 ± 1023.4	<0.01^d^

**Follow-up data**	**RVIAT (*n* = 786)**	**MS (*n* = 779)**	***P* value**

Completeness of follow-up, n (%)	98.6%	97.7%	0.16^a^
Duration of follow-up, median [IQR], months	32.83 [24.63, 42.98]	40.90 [27.90, 49.10]	<0.001^e^
Readmission due to pulmonary complications within postoperative 6 months^f^ (%)	49 (6.2)	65 (8.3)	0.06^a^
Parent- or patient-reported satisfactory score, mean ± SD	9.21 ± 0.06	6.98 ± 1.17	<0.001^d^

a *P*-value was generated using the McNemar test.

b One patient underwent reoperation for bleeding.

c Of the 7 patients, 1 underwent a reoperation due to bleeding and the other 6 underwent reoperations due to pericardial effusion within 2 months postoperatively.

d *P*-value was generated using the pairwise *t*-test.

e *P*-value was generated using the pairwise Wilcoxon test.

f Pulmonary complications included pleural effusion, atelectasis, pneumothorax, and recurrent pneumonia.

AXC: aortic cross-clamping; CICU: cardiac intensive care unit; CPB: cardiopulmonary bypass; IQR: interquartile range; NA: not available; RVIAT: right vertical infra-axillary thoracotomy; SD: standard deviation; US: United States; VSD: ventricular septal defect.

### Learning curve analysis

**Figure 3.**
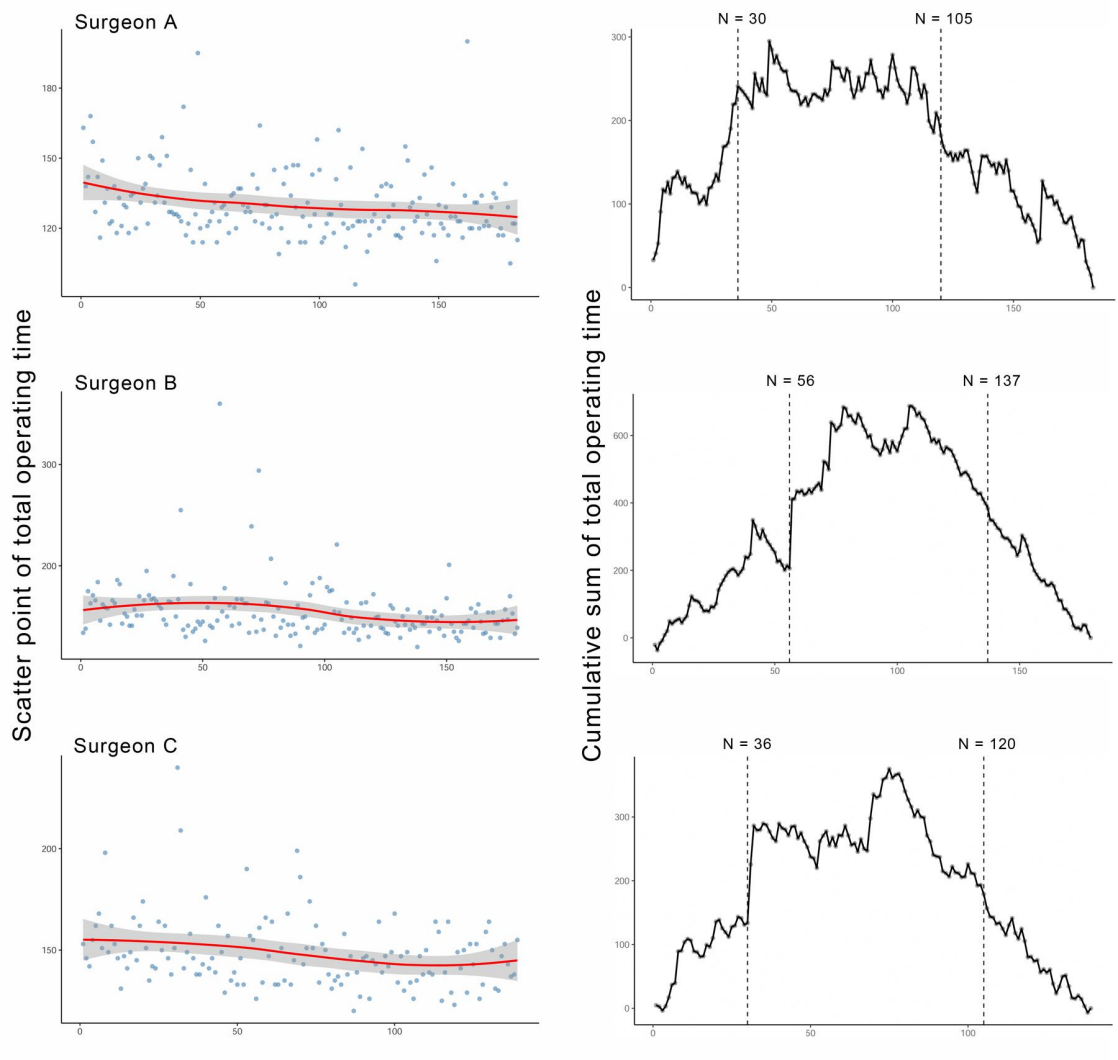
RA-CUSUM analysis of the total operating time for 3 surgeons. For surgeon A, the learning phase, plateau, and master phase were cases 1–30, cases 31–105, and cases 106–139. For surgeon B, the learning phase, plateau, and master phase were cases 1–56, cases 57–137, and cases 138–179. For surgeon C, the learning phase, plateau, and master phase were cases F1–36, cases 37–120, and cases 121–183. RA-CUSUM: risk-adjusted cumulative sum.

After controlling for the baseline characteristics, the learning curve for the RVIAT procedure for the total operating time was observed in all 3 surgeons (**Figure 3**). After performing 30, 56, and 36 cases respectively, the 3 surgeons entered a plateau. Subsequently, with the breaking points estimated at 105, 137, and 120 cases, the total operating time was found to decrease, indicating an attainment of technical proficiency. Specifically, there were no differences in perioperative outcomes for each of the 3 surgeons when stratified by the inflexion points (Supplementary Table S4).

## DISCUSSION

Our data demonstrate several key points: (i) RVIAT was associated with shorter length of postoperative hospital stay, decreased hospitalization cost, and better cosmesis without increasing surgical risks. This procedure appeared to be performed for all paediatric patients across the size spectrum and different VSD subtypes; and (ii) a mean of approximately 56 cases may be required for conquering the early learning phase of RVIAT.

Unlike the prior studies [e.g. small sample sizes^5^^,^^7^^,^^8^^,^^10^ or lack of comparative data^6^^,^^9^^,^^16^, one strength of this study is that we use a large cohort to compare 4-year outcomes of RVIAT versus MS for VSD closure in a controlled manner, which provides evidence-based data to support the application of RVIAT. The well-balanced demographics between subcohorts suggest that the perioperative outcome differences observed were related to the operative approach rather than to the inherent patient characteristics. Significantly reduced duration of postoperative hospitalization with decreased cost for health care is noteworthy, indicating that faster recovery can be anticipated using the RVIAT approach.

Several explanations for quicker recovery in the RVIAT group warrant discussion. First, intercostal nerve block, careful use of a rib spreader for exposure, and the muscle-sparing nature of this approach are helpful for reduced pain and discomfort postoperatively. Moreover, given that no sternal healing needs to occur, patients are encouraged to augment physical activity as tolerated, which may contribute to the reduced duration of chest tube drainage. Second, strategical stay sutures for optimal exposure can avoid extra manipulations by assistants and decrease the risk of surgical injuries. Third, one-lung ventilation can guarantee excellent visualization and facilitate direct central cannulation. This ventilatory strategy helps to avoid femoral bypass, which potentially leads to femoral vessel injury and affects postoperative recovery. These data have implications for advocation of VSD repair through the RVIAT approach with regard to optimizing resource utilization around surgical care.

The RVIAT approach for VSD closure can be performed with significant cosmetic benefits without increased surgical risks compared with the MS approach. A strong desire of the family for the pursuit of cosmetics is an important driving force for the implementation of minimally invasive procedures. Unsurprisingly, the hidden scar underneath a resting arm well satisfies the parents’ urgent demand for improved cosmesis. In addition, the absence of a thoracic deformity is another important reason for higher satisfaction because the sternum healed with bony prominences or asymmetry in a considerable number of patients in the MS group. Importantly, no postoperative morbid event evaluated was higher in the RVIAT group than in the MS group. Neither did we observe an increase in right-sided pulmonary complications within 6 months postoperatively.

The choice of the RVIAT approach for VSD closure depends mostly on the surgeon’s preference and surgical expertise. Despite repeated evidence of the benefits of a minimally invasive approach in congenital heart surgery, there are still many challenges to widespread applicability of RVIAT for VSD closure. Many of these challenges are rooted in an understanding of learning curves and the importance of increasing learning exposure. Results from prior studies^12^^,^^17–20^ have indicated that expanding a minimally invasive approach to repair more complex cardiac lesions involves a certain learning curve. Our findings expand these works by first demonstrating a learning curve effect for cross-clamping time in RVIAT for VSD closure.

The operative surgeons included in RA-CUSUM analysis, although experienced in the MS approach, initially had limited proficiency in minimally invasive procedures. Establishment of the standard of procedure is helpful in terms of how to effectively perform the RVIAT procedure and likely contributed to a favourable learning curve and can likely be translated to other high-volume congenital heart surgery programs. We used the total operating time as a metric to analyse the learning curve effect, given the following considerations: (i) VSD is generally considered to be a benchmark procedure wherein exposure is the key determinant of success. Un-ideal exposure and unfamiliarity with the RVIAT approach complicate the procedure, which may result in a residual defect or injury to the conduction system potentially due to the technical mistakes. Less total operating time, to some extent, indicates that the surgeon obtains more familiarity with the exposure and gains technical expertise for this approach. Plausibly, there is a high likelihood that some potential technical mistakes could be avoided and better results can be expected. (ii) Longer bypass time may also be associated with increased risk of kidney or cardiac injury, which affects the postoperative recovery. (iii) Given the multicentre nature of this study, we did not use other major morbidities (e.g., ventilations times, intensive care unit stay), which may be biased by the different protocols in different centres. Taken together, total operating time appeared to be a useful metric as a surrogate of some morbidities of interest to perform the learning curve analysis.

On the other hand, when talking about a learning curve, a conclusion cannot be made based on a single outcome (e.g., total operating time). And no clear definition exists of what variables exactly constitute a learning curve. Thus, we further compared 2 groups based on the outcomes of RA-CUSUM analysis on total operating time for each of the 3 surgeons: cases before the inflection point versus the rest. Such comparisons demonstrated no difference in postoperative complications, suggesting that more total operating time during the learning phase is acceptable to achieve proficiency without compromising procedural safety. Opponents against minimally invasive approach in congenital heart surgery generally believe that the poor exposure would add to procedural complexity, prolong operation duration, and reduce the margin of error in case complications arise^21^. Our study is partially a departure from these opinions and provides evidence for supporting centres to expose surgeons to an adequate number of procedures to improve expertise.

### Limitations

Although we attempted to minimize the bias using propensity score matching, some confounding factors may influence the outcomes such as individual surgeon experience and era effect. Selection bias may still exist; for example, some surgeons may prefer to use the RVIAT approach for VSD in patients with favourable anatomy, especially during the early period. Considering that (i) the cases were consecutively included and (ii) there was a case mix of VSD with different subtypes and anatomies, it is possible that the distribution of VSD with difficult anatomies (e.g., defect obscured by tricuspid valve tissue or chordal attachments) across the learning period for the RVIAT may influence the cut-off. Although it is unclear whether the results derived from 3 experienced surgeons from the 2 high-volume institutions are generalizable, the acceptable learning curve warrants widespread studies to confirm the feasibility and safety of the RVIAT approach for VSD closure. This series trended towards older patients, and precise information regarding pulmonary hypertension was not available so there was a possibility that patients >12 months old may have a small restrictive defect that potentially confounded our findings. Finally, longer follow-up is required to evaluate whether the skeletal development of the patients in the RVIAT group is perfect because the involved rib usually remains thin when followed up to the teen years and beyond.

## CONCLUSION

This study attests to the utility of the RVIAT for surgical repair of VSD that has advantages in cosmesis, quick postoperative recovery, and economic profits compared with MS. Exposure to an adequate number of RVIAT procedures is crucial to gaining proficiency and would not compromise safety.

## Supplementary Material

ivaf153_Supplementary_Data

## Data Availability

The data underlying this article will be shared on reasonable request to the corresponding authors.
