# Supplementary material for: Right vertical infra-axillary thoracotomy for surgical repair of paediatric ventricular septal defect: a propensity score matched cohort study
Source: Interdiscip Cardiovasc Thorac Surg. 2025 Jun 27;40(8):ivaf153. doi: 10.1093/icvts/ivaf153 (PMC12451698; doi:10.1093/icvts/ivaf153)
Supplement: ivaf153_Supplementary_Data [file ivaf153_Supplementary_Data.zip › Legend for Supplemental Video 1.docx]

**Supplemental Video 1.** This film made up of 3 parts shows the RVIAT approach for surgical repair of ventricular septal defect and pulmonary stenosis in a 4-month boy with the weight of 7.8 kg. RVIAT: right vertical infra-axillary thoracotomy.
