# Supplementary material for: Right vertical infra-axillary thoracotomy for surgical repair of paediatric ventricular septal defect: a propensity score matched cohort study
Source: Interdiscip Cardiovasc Thorac Surg. 2025 Jun 27;40(8):ivaf153. doi: 10.1093/icvts/ivaf153 (PMC12451698; doi:10.1093/icvts/ivaf153)
Supplement: ivaf153_Supplementary_Data [file ivaf153_Supplementary_Data.zip › SupplementaryTable.docx]

**Supplemental Table 1. Standard of procedure in RVIAT approach**

| **Step 1**  (Position) | After endotracheal intubation, patients are positioned in a 45° left lateral position with the arm elevated above the head and kept in an arm rest. |
| --- | --- |
| **Step 2**  (Preoperative Marking) | The anterior, posterior axillary line and the 4th as well as 7th intercostal space are marked with a pen. |
| **Step 3**  (Muscle-sparing Approach) | After retraction of serratus anterior and latissimus dorsi, a thoracotomy vertical incision is made in the 4th intercostal space. If the patient weight is less than 25 kg, one rib spreader is used; otherwise, two rib spreaders (placed in a cross position) are used. Additionally, a second 0.5-cm vertical incision was made at 7th intercostal space which is used for IVC cannula tunnel later, and is also used for the chest tube. |
| **Step 4**  (Exposure) | (1) Thymus is partially or completely resected for optimal visualization.  (2) Pericardium is opened 1.5-2 cm anterior to the phrenic nerve.  (3) Strategical placement of 5 stay sutures, including 3 along the right margin and 2 along the cephalic margin of the pericardium.  (4) A small wet gauze is placed between the lung and pericardium for lung retraction and protection if one-lung ventilation is not performed. |
| **Step 5**  (Purse Strings and Snares) | (1) Order of purse strings: SVC (purse string in a flat oval shape), aorta, and IVC.  (2) SVC snare is placed in a routine fashion as is placed in the MS approach; renal pedicle clamp is used to facilitate the placement of IVC snare. |
| **Step 6**  (Ligation of PDA before CPB if existed) | The assistant uses a long vessel forceps to pull the main pulmonary artery towards the right ventricular outflow tract for better exposure, and then the PDA is ligated. |
| **Step 7**  (Direct Central Cannulation) | After all the purse strings and snares are placed, direct central cannulation will be performed:  (1) Aortic cannulation: the assistant uses a long vessel forceps to pull the aorta towards the left ventricular outflow tract to assist cannulation.  (2) SVC cannulation: a right-angled metal tip cannula is used for cannulation.  (3) IVC cannulation: a malleable cannula is used; a right-angle clamp is used to adequately lift up the IVC from the bottom of the vessel to facilitate the cannulation; the IVC cannula is tunneled through the skin at the site of a 0.5 cm-incision in the 7th intercostal space. |
| **Step 8**  (Exposure of the Defect) | (1) Perimembranous VSD: after right atriotomy, three traction sutures were placed for better exposure of VSD, including two placed at 12 and 3 o’clock of the TV annulus and the other one pledget stitch placed at 1 o’clock of the defect and 2 cm away from the defect rim on the right side of the ventricular septum.  (2) Doubly-committed subarterial VSD: pulmonary trunk is longitudinally opened, and one stay suture is placed at each side of the pulmonary trunk incision for traction. |
| **Step 9**  (Defect Closure) | (1) Perimembranous or Doubly-committed subarterial VSD is closed with a pericardial patch using a running suture.  (2) Inlet muscular VSD (< 3 mm) is directly closed using interrupted pledget stitch. |
| **Step 10**  (Concomitant Maneuver) | (1) Obstruction of right ventricular outflow tract is relieved if existed.  (2) ASD is closed if existed. |

IVC: inferior vena cava; SVC: superior vena cava; MS: median sternotomy; PDA: patent ductus arteriosus; CPB: cardiopulmonary bypass; VSD: ventricular septal defect; TV: tricuspid valve; ASD: atrial septal defect.

**Supplementary Table 2. Parent- or patient-reported cosmetic satisfaction.**

|  | Satisfactory (2 points) | Acceptable (1 point) | Unsatisfactory (0 point) |
| --- | --- | --- | --- |
| Displeasing scars |  |  |  |
| Pectus excavatum |  |  |  |
| Pectus carinatum |  |  |  |
| Asymmetrical thorax |  |  |  |
| Asymmetry of the breast |  |  |  |

**Supplementary Table 3. Detailed information regarding the complications**

|  | RVIAT (n = 797) | MS (n = 797) | P value |
| --- | --- | --- | --- |
| Any admissions due to pneumonia after operation, n (%) | 49 (6.2) | 65 (8.2) | 0.15 |
| Pneumonia in 3 months, n (%) | 8 (1.0) | 10 (1.3) | 0.64 |
| Recurrent pneumonia, n (%) | 7 (0.9) | 6 (0.8) | >0.99 |
| Pectus excavatum, n (%) | 0 (0.0) | 1 (0.1) | > 0.99 |
| Pectus carinatum, n (%) | 0 (0.0) | 3 (0.4) | 0.25 |
| Displeasing scar, n (%) | 3 (0.4) | 21 (2.6) | < 0.001 |
| Assymetrical thorax, n (%) | 3 (0.4) | 28 (3.5) | < 0.001 |
| Assymetrical shoulders, n (%) | 2 (0.3) | 1 (0.1) | > 0.99 |
| Imbalancing steps, n (%) | 2 (0.3) | 5 (0.6) | 0.45 |
| Abnormal gait, n (%) | 1 (0.1) | 5 (0.6) | 0.22 |

**Supplementary Table 4. Comparison in the primary endpoints before and after the plateau of the learning curve in three surgeons.**

|  | Before Plateau | After Plateau | P value |
| --- | --- | --- | --- |
| Surgeon A | Case 1-105  (n = 105) | Case 106-139  (n = 34) |  |
| Death (%) | 0 (0) | 0 (0) | NA |
| Rhythm disturbances requiring temporary cardiac pacing (%) | 1 (1.0) | 0 (0) | 1.00 |
| Residual significant VSD (%) | 0 (0) | 0 (0) | NA |
| Reoperation due to pericardial effusion or bleeding (%) | 1 (1.0) | 0 (0) | 1.00 |
| Surgeon B | Case 1-137  (n = 137) | Case 138-179  (n = 41) |  |
| Death (%) | 0 (0) | 0 (0) | NA |
| Rhythm disturbances requiring temporary cardiac pacing (%) | 0 (0) | 0 (0) | NA |
| Residual significant VSD (%) | 0 (0) | 0 (0) | NA |
| Reoperation due to pericardial effusion or bleeding (%) | 0 (0) | 0 (0) | NA |
| Surgeon C | Case 1-120  (n = 120) | Case 121-183  (n = 63) |  |
| Death (%) | 0 (0) | 0 (0) | NA |
| Rhythm disturbances requiring temporary cardiac pacing (%) | 1 (0.8) | 0 (0) | 1.00 |
| Residual significant VSD (%) | 0 (0) | 0 (0) | NA |
| Reoperation due to pericardial effusion or bleeding (%) | 0 (0) | 0 (0) | NA |

VSD: ventricular septal defect; NA: not available.
